# Investigation of Outbreaks Complicated by Universal Exposure

**DOI:** 10.3201/eid1811.111804

**Published:** 2012-11

**Authors:** Alma Tostmann, Teun Bousema, Isabel Oliver

**Affiliations:** Health Protection Agency, Gloucester, UK (A. Tostmann, I. Oliver);; European Centre for Disease Control, Stockholm, Sweden (A. Tostmann);; London School of Hygiene and Tropical Medicine, London, UK (A. Tostmann, T. Bousema);; Radboud University Nijmegen Medical Centre, Nijmegen, the Netherlands (T. Bousema);; and Bristol University, Bristol, UK (I. Oliver)

**Keywords:** outbreak, infectious disease, epidemiology, public health, bacteria, viruses

## Abstract

Outbreaks in which most or all persons were exposed to the same suspected source of infection, so-called universal exposure, are common. They represent a challenge for public health specialists because conducting analytical studies in such investigations is complicated by the absence of a nonexposed group. We describe different strategies that can support investigations of outbreaks with universal exposure. The value of descriptive epidemiology, extensive environmental investigation, and the hypothesis-generation phase cannot be overemphasized. An exposure that seems universal may in fact not be universal when additional aspects of the exposure are taken into account. Each exposure has unique characteristics that may not be captured when investigators rely on the tools readily at hand, such as standard questionnaires. We therefore encourage field epidemiologists to be creative and consider the use of alternative data sources or original techniques in their investigations of outbreaks with universal exposure.

## Abstract 2

Maximizing the information collected about aspects of the exposure can support investigations of disease outbreaks complicated by universal exposure.

Infectious disease outbreaks in which most or all persons are exposed to the same potential source of infection, so-called universal exposure, are common. They can occur in a range of settings (e.g., healthcare, community) and involve various organisms and vehicles. When outbreaks with universal exposure are investigated, it is difficult to explore the possible association between illness and that specific exposure by using conventional epidemiologic and environmental approaches.

Universal exposure complicates the analysis of epidemiologic studies because of the absence of a nonexposed group, which makes it difficult to test the hypotheses under investigation. This article provides an overview of options for outbreak investigations in which the exposure to the suspected source of infection is universal. We conducted a thorough literature search to collect articles on outbreak investigations in which analytical techniques were used that could be helpful in a situation of universal exposure. In addition, the European Programme for Intervention Epidemiology Training (EPIET) network and the European Programme for Intervention Epidemiology Training Alumni Network were asked to provide published or unpublished outbreak reports of outbreaks with universal exposure

## Outbreaks with Universal Exposure

Biological plausibility of the suspected source is an essential link in the chain of evidence. In a recent outbreak of campylobacteriosis after a wedding dinner in southwestern England, all guests had eaten the same appetizer, chicken liver parfait. This item was the suspected source of the outbreak. The increase in campylobacter outbreaks linked to poultry liver parfait in the United Kingdom appears to be associated with intentional undercooking of the poultry livers (*1*). In this outbreak investigation, we were unable to compare the consumption of this appetizer between case-patients and non–case-patients because exposure was universal. Apparently, almost all guests had completely finished the single portion they were given, and we could not investigate a dose-response association. No food sample was available for microbiologic testing, which further reduced the options to provide evidence for this biologically plausible source of the campylobacter outbreak.

In a salmonellosis outbreak in southwestern England in 2011, illness was linked to the consumption of pork meat served at a hog roast (barbecued pig) in a small town. Eight laboratory-confirmed cases of infection with *Salmonella enterica* serotype Typhimurium of the same genetic subtype were identified through routine surveillance. All case-patients had visited the town on the same day, and an epidemiologic study was conducted to test several hypotheses about the potential source of the outbreak. However, almost all persons interviewed had consumed pork from the hog roast, making it impossible to compare pork consumption of case-patients and noninfected persons. The circumstantial evidence pointed toward pork meat being the source of the outbreak because pork can be a source of *S. enterica* ser. Typhimurium (*2*). It was the only common exposure among the case-patients with confirmed salmonellosis, none of the other potential risk factors (including other foods or drinks consumed in the village on that day) were linked to illness, and no other common exposures were identified. Unfortunately, no leftover pork was available for microbiologic testing.

## Options for Investigations Involving Universal Exposure

Many public health specialists will recognize outbreak situations such as those described above: a hypothesis based on biological plausibility cannot be confirmed because of the absence of microbiologic evidence and the difficulties of obtaining strong epidemiologic evidence as a result of universal exposure. These situations require creativity to maximize the information that is available from the exposure.

A key step in any outbreak investigation is the hypothesis-generation phase (*3*). In-depth interviews with the first case-patients and other stakeholders involved are essential to collect sufficient background information to generate the hypotheses. In this phase, whether universal exposure will potentially be an issue may become evident. This gives the investigators the opportunity to decide what additional aspects of the exposure could be explored in the analytical study. It is tempting, but limiting, to rely only on standard techniques and trawling questionnaires (series of open questions covering activities before onset of illness) because each outbreak is characterized by unique exposure aspects that may not be captured when one relies exclusively on the tools that are readily at hand.

Possible approaches to investigating outbreaks complicated by universal exposure include robust descriptive epidemiology, optimization of the dose-response analysis, and in-depth exposure analysis to determine whether the alleged universal exposure is indeed uniform exposure. Exposures that seem universal initially may in fact not be universal on in-depth analysis of additional aspects of the exposure. Most techniques described in this article were used in outbreaks not complicated by universal exposure, but they can be applied to outbreaks with universal exposure.

### Strong, Descriptive Epidemiology

Well-presented descriptive epidemiologic characteristics can provide sufficient evidence for a suspected source in an outbreak investigation. Outbreak-associated cases are usually described by time, place, and person. Maximizing the information that is available by analyzing different aspects of time, investigating strong geographic clustering, or focusing on the odd, but highly informative, outliers can help build evidence toward the suspected source of infection.

#### Time: Exposure at Peak Times

The time of eating may be a relevant aspect of exposure in outbreak investigations in which cooking procedures differ over time. For example, at a barbecue, the meat that was served in the beginning, at the end, or at the peak time of preparation may have been undercooked. This variation can also occur at a dinner in which several servings of the same dishes were served consecutively. The data analysis can be stratified by comparing those who ate early with those who ate later or by time of the servings. A difference in attack rates between these subgroups can be an indication for the exposure as source of the outbreak.

In addition, high sales pressure at peak times can potentially result in the sale of undercooked meat products. In 2009, an outbreak of *S. enterica* ser. Enteritidis was linked to a kebab shop in West London (*4*). Because dishes were consumed simultaneously, associations between various food items and illness could not be disentangled. The investigators used till receipts to identify the peak times at the food outlet and found that customers who had consumed chicken kebab during peak times were more likely to becomet ill. A plausible explanation is that during peak times the rotisserie chicken was undercooked.

#### Place: Strong Geographic Clustering

Geographic clustering can be informative in identifying the source of an outbreak and can be established by mapping case-patients by place of residence. The cholera outbreak investigations of Dr John Snow hold some practical examples of field epidemiology that are still being used today. The strong geographic clustering of cholera cases in central London in 1854 implicated the Broad Street pump as a suspected source of infection (*5*).

In waterborne outbreaks, the pathogen may not always be isolated from the water, because contamination may have ended when the investigation starts. There may still be ways to collect water samples from the time of the outbreak (e.g., ice cubes, looking for water dead ends such as fire hydrants), but generally such investigations rely heavily on epidemiologic evidence rather than confirmation by microbiologic testing.

Tap water is a typical source of universal exposure. However, drinking water is usually distributed in different supply zones, with the result that the exposure is not necessarily universal. After a large outbreak of *Giardia* infections with 1,300 laboratory-confirmed cases in Norway in 2004, geographic clustering of cases in 1 water distribution area pointed toward the water supply serving Bergen city center as the potential source. The attack rate was ≈18 times higher in this supply zone than the rate in the other supply zones combined (*6*). In addition, within a supply zone, the distance from the suspected point of pathogen introduction can be associated with decreasing attack rates. Thus, even if all study participants reside in the same water supply zone, geographic location within the zone could be used to calculate distance attack rates.

Distance attack rates have been applied to an outbreak of *Coxiella burnetii* infections in the Netherlands. Living near ruminant farms is a risk factor for infection with *C. burnetii,* the causative agent of Q fever. In May 2008, 96 cases of Q fever occurred in a small urban area in the Netherlands, and a common source was suspected (*7*). Several ruminant farms were located in the area, and a geographic information system was used for the investigation, taking into account the postal codes of the patients and the farms. Distance-related attack rates and relative risks for increasingly larger ring buffers were calculated for each of the large farms in that area, highlighting the usefulness of spatial analysis in analyzing a cluster of cases.

Another example is that if an outbreak is confined to a single geographic location and 2 products are suspected as sources of the outbreak, one with a national and one with a local distribution, the product with the local distribution could be considered the most likely source. Also, when nearly universal exposure to a common food item (such as chicken) is presumed, more heterogeneous exposures may be defined if exposure is defined on the basis of the origin of the food item, e.g., the different suppliers or processors.

#### Person: Focusing on Outliers

The historical example of the cholera outbreak in London not only illustrates the value of spatial analysis, but also the value of outliers in an outbreak investigation. Dr Snow found that nearly all persons who died of cholera lived within a short distance of the Broad Street pump, except for 2 patients who lived at the other end of town, an elderly woman who had not been near Broad Street for many months and her niece. It appeared that the woman had a bottle of Broad Street pump water delivered to her doorstep each day, and this had occurred on the day before she fell ill. The niece drank from the same water when visiting her aunt (*5*).

Early in 2011, 14 persons from southern France, with laboratory-confirmed cases of *S. enterica* ser. Enteritidis infection, had eaten from a common lunch at a hunting party. A cohort study among party attendees showed that the only food item that could explain all cases was wild boar meat. However, almost all of the 50 study participants had consumed wild boar meat. The outliers in this study were 5 participants who had only consumed leftovers: 4 became ill and they all had eaten boar meat. The person who did not fall ill ate only blood pudding. Despite of the absence of a nonexposed group in the analysis of wild boar meat consumption, the results strongly suggested that the wild boar meat was the source of infection (*8*).

Another example of the potential role of the outlier is illustrated by the investigation of an unusual and large outbreak of hemolytic uremic syndrome (HUS) and diarrhea caused by Shiga toxin–producing *Escherichia coli* (STEC) O104:H4, which occurred in Germany in 2011 (*9*,*10*). A satellite outbreak in France, which could be considered as an outlier of the large outbreak in Germany, provided the investigation with central clues that led to the identification of fenugreek sprouts as the vehicle of this outbreak. In June 2011, an outbreak of HUS associated with STEC O104:H4 occurred in Bordeaux, France, and the *E. coli* isolates were genetically related to the strain found in the German outbreak (*11*,*12*). This was the first outbreak outside Germany in which infection was not related to travel to Germany. Preliminary investigations determined that case-patients had attended an open day at a community center where a cold buffet with vegetables was served. Because sprouts were already suspected as a vehicle in the German outbreak, the French investigators examined sprout consumption in detail. Three types of sprouts were served at the buffet: mustard sprouts, rocket sprouts, and fenugreek sprouts. In the analysis, sprouts were identified as a risk factor for illness, and multivariate analysis showed that the fenugreek sprouts were the most likely vehicle for transmission.

### Optimizing Dose-Response Analysis

Assessing a dose-response relationship is a well-known analytical tool for identifying the source of an outbreak. An increased risk for disease or increased severity of symptoms with increased exposure dose indicates such a dose-response relation between the suspected source and the illness. Normally, the reference category in a dose-response analysis is the unexposed group, but in outbreaks with universal exposure, the lowest exposure category can be used as a reference group instead.

Exposure levels should be considered in the design phase of the analytical study to ensure that dose response can be evaluated in the analysis stage. Questions about quantities can aim at either the exact quantity (e.g., the number of glasses tap water per day, the number of canapés eaten at a wedding reception) or at more qualitative levels of exposure (e.g., a bite, half a portion, 1 portion, >1 portion). How best to assess dose depends on the type of food investigated and the circumstances under which it was consumed.

In cohort studies, attack rates and relative risks can be calculated for each level of exposure. In case-control studies, the proportion of persons exposed to each level of exposure can be calculated for case-patients and controls and odds ratios calculated for each level of exposure. The group with the lowest exposure will serve as reference group. An increase in attack rate (cohort study) and effect size (risk ratio or odds ratio) with increasing dose, supported by a statistical test for trend, implicates a dose-response relation.

Two examples illustrate investigations of outbreaks linked to tap water in which the reference group consisted of those with the lowest exposure rather than an unexposed group. An outbreak of campylobacteriosis in Norway was associated with consumption of tap water (*13*). A cohort study showed that the attack rate increased from 40% for those who had drunk 1–3 glasses per day (the reference group) to ≈65% for those who had drunk >6 glasses of tap water per day. In the previously mentioned outbreak of giardiasis in Bergen, Norway, a case–control study showed that persons who drank the largest quantity of water had the highest risk for illness; the strength of the association (odds ratio) increased with increased water consumption (*6*). Assessing a dose-response relationship may be more complicated if the exposure cannot be readily quantified or if the vehicle is heavily contaminated.

### Exploring Individual Components of Exposure: Ingredient-based Analysis

The recent large outbreak of STEC in Germany demonstrated that hidden or marginal ingredients such as bean sprouts, sometimes solely used for decoration of a dish, can cause large outbreaks (*9*,*10*). Even though this outbreak was not complicated by universal exposure, it demonstrates valuable lessons for field epidemiologists confronted with universal exposure. It can be highly informative to explore the individual components of an exposure, for example, the actual ingredients of a specific dish as reported by those cooking it rather than the dish or products consumed as reported by the consumer, who may be unaware of the less obvious ingredients.

Although investigation of a foodborne outbreak may reveal that all persons have eaten the same dish, they may not have eaten all ingredients from their plates. An appetizer may include some edible decoration, such as bean sprouts, that some will put aside, a salad may contain different ingredients that not all like to eat, or a seafood cocktail may hold different species from which a person may select only a few. A thorough environmental investigation is therefore essential.

The investigation of the STEC outbreak in Germany consisted of several studies that followed up on each of the findings. Since the earlier studies had not identified a single source of infection, the outbreak control team conducted a cohort study at a restaurant because several case-patients had dined at that restaurant during May 12–16, 2011 as part of a group (*9*,*10*). A review of the booking notes identified 10 groups (total 177 persons) that had eaten in the restaurant during that period. Thirty-one of the 168 customers who were interviewed had become ill, and HUS developed in 8 persons. When the role of the separate ingredients used for each of the dishes was investigated, sprouts were the only food item independently associated with illness.

An outbreak of gastrointestinal illness at a conference in Wales, United Kingdom, in 1991 was associated with the seafood cocktail, which contained precooked mussels, prawns, and cockles (*14*). Although consumption of the cocktail was nearly universal, only three fourths of those who ate the cocktail had eaten the mussels, the only component that was independently associated with illness.

A combination of ingredient based and dose-response analysis established the relation between exposure of methomyl-contaminated salt, and gastrointestinal illness in a Thai restaurant in the United States in 1999; an ultimate example of universal exposure because all restaurant clients had consumed some salt as a consequence of the preparation process (*15*). Because no specific dish could account for most cases, the association with specific ingredients was investigated. The exposure to salt was estimated by using the amount of salt in the recipe for each dish, the quantity that was consumed in each dish, and the quantity of salt that was added by respondents after the meal had been served. When the ingredient was quantified is this manner, the risk for illness was convincingly associated with the total quantify of salt consumed.

In a massive multistate salmonellosis outbreak (≈1,500 cases), the most likely source was red salsa made from tomatoes and raw jalapeño peppers (*16*). Two clusters of outbreak-associated cases (patrons from 2 different restaurants) were investigated. To produce the salsa served during the outbreak period, 1 restaurant used ≈25 boxes of tomatoes of various brands, whereas only 1 box of jalapeño peppers was used. When exposure to ingredients (tomatoes, jalapeño peppers), rather than exposure to the food product (salsa), was considered, the jalapeño peppers were the only common exposure between the 2 restaurants; contaminated peppers were eventually traced back to a single farm in Mexico. In this example, the combination of ingredient-based analysis and geographic aspects of the suspected source (e.g., the origin of the product, distribution area, packing facilities) provided key information in the source-finding process.

These outbreak investigations show that individual components of a mixed dish can be identified as the vehicle of infection. Scrutinizing associations with individual ingredients may reveal that exposure that appears universal is, in fact, heterogeneous. Caveats of ingredient-based analysis include the possibility of cross-contamination between different ingredients and that persons may have difficulties in recalling what ingredients they have consumed. These possibilities can be overcome by talking to the cook or food producers.

### Use of Different Data Sources to Analyze Exposure

Alternative sources of information can be used to support or refute possible hypothesis generated by using standard outbreak investigation methods. Examples include the use of till receipts (*4*) or meteorologic data (*7*,*17*).

In the Q fever outbreak investigation in the Netherlands described previously, the team used meteorological data to further support their hypothesis (*7*). The geographic and veterinary data pointed toward a large dairy goat farm that was located northeast from the town. Meteorologic data gave further support because it showed that there were many days with predominant easterly winds that could have taken contaminated dust particles to the persons living southwest of the farm.

In an outbreak of Q fever in a town in southeastern England, atmospheric dispersion modeling (using meteorologic data, including ground wind speeds and direction) was used to investigate potential airborne transport of *C. burnetii* between several suspected sources and the Q fever case-patients in that town (*17*). Three high-risk farms were identified by the veterinary investigation. Dispersion modeling showed that air from each of these suspected farms may have exposed the town to the bacteria at some point over the study period and none of the suspected farms could be ruled out as a potential source. Nevertheless, this approach shows the potential of using nonconventional data sources in outbreak epidemiology.

## Discussion

Outbreaks with universal exposure represent a challenge for public health specialists because conventional investigational approaches may be insufficient to reach informative conclusions. Robust descriptive epidemiology, a thorough environmental investigation, and the hypothesis-generation phase become even more essential. In-depth interviews with the initial patients can be highly informative in determining whether a risk for universal exposure exists. If universal exposure is suspected, additional aspects of that exposure should be explored because exposures that initially seem universal may in fact not be universal after all when studied in more detail.

Although a balance must be found between the quality and quantity of questions in a questionnaire, once a hypothesis is generated on the basis of initial interviews, more detailed information about the exposure to likely sources, such as time and quantity of exposure, can provide useful additional information to support or refute the hypothesis. Universal exposures are often ignored if another item is significantly associated with illness. Investigators need to at least consider the universal exposures before accepting a statistically significant association. It remains, however, essential to rule out other potential exposures before accepting that the universal exposure was the cause of the outbreak. Because of the difficulty in obtaining conclusive findings, studies of outbreak investigations complicated by universal exposure are less likely to be published and less likely to result in recommendations for public health measures to prevent continuation or recurrence of exposure. We hope that this article provides readers with several tools to overcome this problem and maximize the effect of outbreak investigations on public health.
